# Inflammatory macrophages can transdifferentiate into myofibroblasts during renal fibrosis

**DOI:** 10.1038/cddis.2016.402

**Published:** 2016-12-01

**Authors:** Xiao-Ming Meng, Shuang Wang, Xiao-Ru Huang, Chen Yang, Jun Xiao, Yang Zhang, Ka-Fai To, David J Nikolic-Paterson, Hui-Yao Lan

**Affiliations:** 1 Li Ka Shing Institute of Health Sciences, Department of Medicine & Therapeutics, The Chinese University of Hong Kong, Hong Kong SAR, China; 2 School of Pharmacy, Anhui Medical University, Anhui, China; 3 Department of Chemical Pathology, The Chinese University of Hong Kong, Hong Kong SAR, China; 4 Department of Anatomical and Cellular Pathology, The Chinese University of Hong Kong, Hong Kong SAR, China; 5 Department of Nephrology and Monash University Centre for Inflammatory Diseases, Monash Medical Centre, Clayton, VIC 3168, Australia

## Abstract

Myofibroblasts play a central role in renal fibrosis although the origin of these cells remains controversial. We recently reported that bone marrow-derived macrophages can give rise to myofibroblasts through macrophage to myofibroblast transition (MMT). However, several important issues remain to be addressed, including whether MMT occurs in human kidney disease and verification of the MMT process through lineage tracing. Biopsies from a cohort of 58 patients with various forms of kidney disease were examined for MMT cells that co-express macrophage (CD68) and myofibroblast (*α*-smooth muscle actin, *α*-SMA) markers. MMT cells were evident in active fibrotic lesions, but were largely absent in acute inflammatory or sclerotic lesions, suggesting that MMT cells contribute to progressive renal fibrosis. Fate-mapping studies in *LysM*^*Cre*^*Tomato* mice identified substantial numbers of Tomato^+^ myeloid cells with F4/80^+^ macrophage phenotype expressing *α*-SMA and collagen I in the unilateral ureteral obstructive model of renal fibrosis, providing direct evidence for the MMT process during the development of renal fibrosis. In addition, MMT cells had a predominant M2 phenotype in both human and mouse renal fibrosis. Finally, selective depletion of myeloid cells via diphtheria toxin in *LysM*^*Cre*^*iDTR* mice largely abolished macrophage infiltration and MMT cells in the obstructed kidney and substantially reduced accumulation of *α*-SMA^+^ myofibroblasts and collagen deposition, revealing a pathogenic role for inflammatory macrophages in MMT and tissue fibrosis. In conclusion, these findings provide substantial new data to support the postulate that macrophages can directly transdifferentiate into collagen-producing myofibroblasts in human and experimental kidney disease.

Myofibroblasts are a subset of activated fibroblasts, characterized by expression of *α*-smooth muscle actin (*α*-SMA), which are the principal cell type responsible for excessive collagen deposition during renal fibrosis that is regarded as a common pathway leading to end-stage renal failure in chronic renal diseases.^1, 2, 3, 4^ However, the origin of myofibroblasts in the fibrosing kidney has remained an issue of debate.^5, 6, 7^ Studies in animal disease models and in cell culture systems have identified several possible origins for the myofibroblasts present in active renal fibrosis, including epithelial–mesenchymal transition (EMT),^8, 9, 10^ endothelial–mesenchymal transition,^11, 12^ local proliferation of resident fibroblasts or pericytes^13, 14^ and circulating fibrocytes.^15, 16^ We have recently described one further potential mechanism: macrophage to myofibroblast transition (MMT).^17^

Evidence for this MMT process is based upon adoptive transfer and bone marrow chimera studies in the mouse model of unilateral ureteric obstruction (UUO) and cell culture studies.^17^ It is proposed that bone marrow-derived macrophages can enter the injured kidney and then transdifferentiate into collagen-producing *α*-SMA^+^ myofibroblasts via TGF-*β*/Smad3 signaling.^17^ However, several important issues remain to be addressed: (i) does MMT occur in human fibrotic kidney disease; (ii) can MMT be confirmed by lineage tracing studies, and (iii) can lineage depletion of macrophages prevent the MMT response and suppress renal fibrosis?

This study addressed these questions by investigating the MMT process in human kidney disease and by using the LysM-Cre myeloid promoter to trace macrophages undergoing MMT, and to determine whether depleting cells of this lineage can suppress renal fibrosis.

## Results

### MMT in human kidney fibrosis

**Figure 1:**
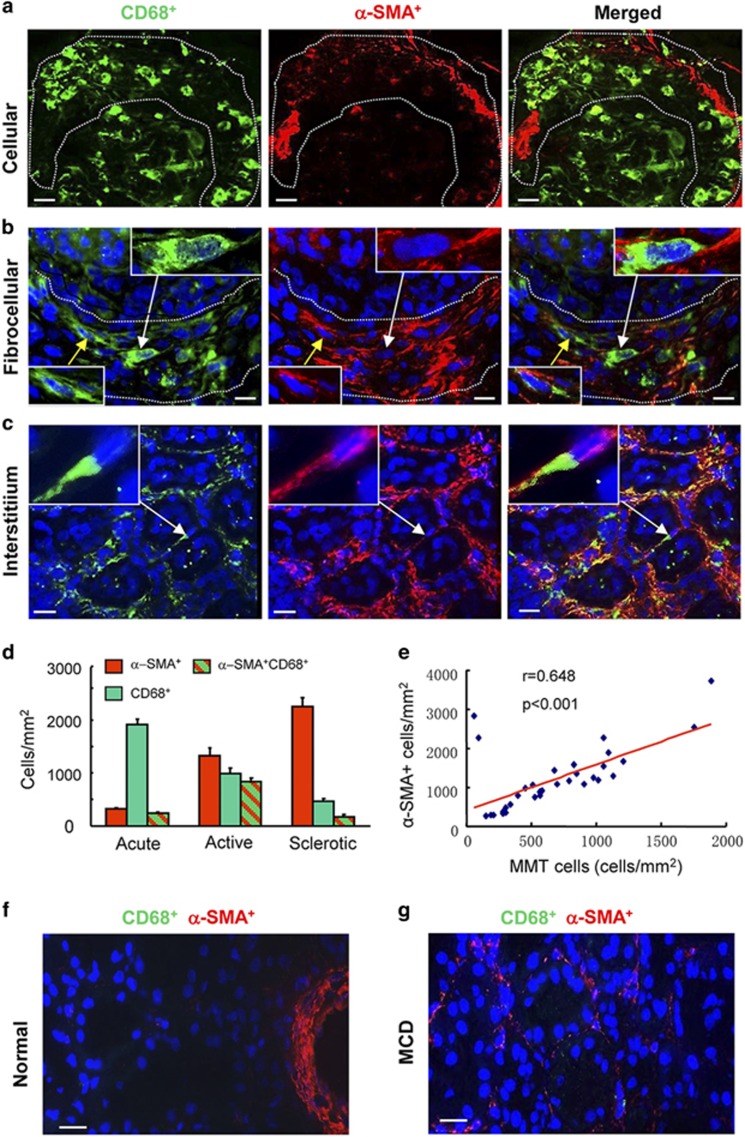
Evidence of macrophage–myofibroblast transition in human kidney disease. (**a**) Results of confocal microscopy indicate that CD68^+^ macrophages (green) and *α*-SMA^+^ myofibroblasts (red) are distinct populations in a case of acute glomerular inflammation featuring a cellular crescent (outlined). (**b** and **c**) The presence of co-expressing CD68^+^*α*-SMA^+^ cells (arrows and insets) can be seen in a fibrocellular crescent (outlined) (**b**) and in an area of interstitial fibrosis in a case of IgA nephropathy (**c**). (**d**) Quantification of CD68^+^ macrophages, *α*-SMA^+^ myofibroblasts and MMT cells co-expressing both markers (CD68^+^*α*-SMA^+^) in acute inflammation (acute, *n*=7), active fibrosis (active, *n*=25) or advanced sclerotic (sclerotic, *n*=14) lesions. (**e**) Spearman correlation analysis of the number of CD68^+^*α*-SMA^+^ cells *versus* total *α*-SMA^+^ myofibroblast accumulation from 32 cases of fibrotic kidney disease. (**f**) Normal human kidney. (**g**) Minimal change kidney disease (MCD). Nuclei are labeled with DAPI (blue). Scale bar, 20 *μ*M

The term 'macrophage to myofibroblast transition' (MMT) describes the overall process by which a macrophage transitions into a myofibroblast, while the term 'MMT cells' refers to individual cells that are in the process of transition with an intermediate phenotype in which they express markers of both lineages. To identify MMT cells in human kidney disease, we sought to detect cells that express both macrophage (CD68) and myofibroblast (*α*-SMA) markers. Biopsy tissues were staged as acute (inflammation with little fibrosis), active (inflammation with active fibrosis) or sclerotic (advanced sclerosis with little inflammation) lesions. In patients with crescentic glomerulonephritis, numerous CD68^+^ macrophages were identified in acute inflammatory lesions such as cellular crescents, but few CD68^+^*α*-SMA^+^ cells were evident (Figures 1a and d). In contrast, a substantial number of CD68^+^*α*-SMA^+^ cells were present in biopsies featuring active fibrosis in fibrocellular crescents and progressive interstitial fibrosis in patients with IgA nephropathy (Figures 1b–d), indicating that MMT accounts for a substantial proportion of the myofibroblast population in active renal fibrosis. Biopsies with advanced sclerosis showed an overall increase in the number of *α*-SMA^+^ myofibroblasts and a reduction in both total CD68^+^ macrophages and in CD68^+^*α*-SMA^+^ MMT cells (Figure 1d). There was a significant correlation between the number of MMT cells and the total *α*-SMA^+^ myofibroblast population in acute and active fibrosis (Figure 1e). In contrast, MMT cells were not detected in normal human kidney or minimal change disease (Figures 1f and g).

**Figure 2:**
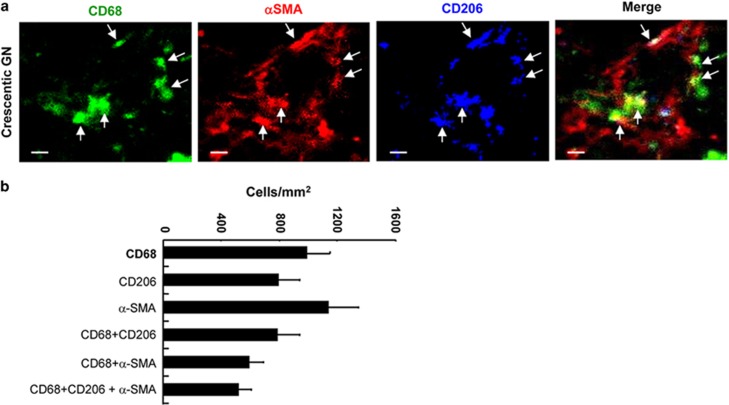
Macrophages undergoing myofibroblast transition have a predominant M2 phenotype in human kidney disease. (**a**) Three-color confocal microscopy identifies triple-stained *α*-SMA^+^CD68^+^CD206^+^ M2-type cells (arrows) in an area of interstitial fibrosis in a patient with crescentic glomerulonephritis (GN). (**b**) Quantitative analysis of the different labeled cell populations in three-color immunofluorescence staining for patients with crescentic GN and IgA nephropathy (*n*=6). Scale bar, 20 *μ*M

We next examined whether MMT cells exhibited an M2 phenotype based on the well-characterized M2 marker, CD206. Three-color confocal microscopy was performed in six cases with active fibrosis. The majority of CD68^+^ macrophages in these cases with active fibrosis co-expressed the CD206 marker. Furthermore, the majority of CD68^+^*α*-SMA^+^ cells expressed CD206, suggesting that it is predominantly M2-type macrophages that undergo MMT in human renal fibrosis (Figure 2).

### Macrophage lineage tracing identifies MMT in experimental renal fibrosis

**Figure 3:**
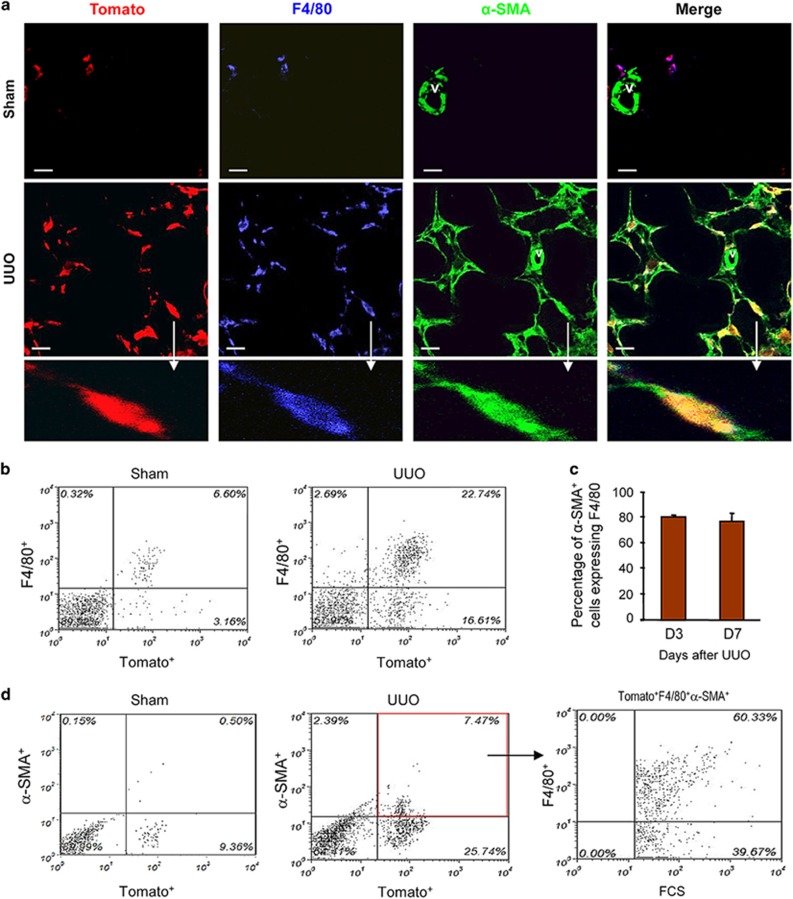
Fate mapping identifies a macrophage origin of MMT cells during renal fibrosis in the obstructed kidney. Groups of eight LysM-Cre/Rosa26-tdTomato transgenic and littermate wild-type mice underwent unilateral ureteric obstruction (UUO) or sham surgery and were killed 7 days later. (**a**) Results of confocal microscopy show co-localization of the Tomato reporter (red) with F4/80^+^ (Green) macrophages, whereas *α*-SMA-expressing vascular smooth muscle cells (v) and tubular epithelial cells lack Tomato expression in the sham-operated kidney. The number of F4/80^+^ macrophages is significantly increased in the day 7 UUO kidney in which more than 90% of macrophage co-express the Tomato reporter. MMT cells derived from the macrophage lineage in the fibrotic kidney are identified as Tomato^+^F4/80^+^*α*-SMA^+^ cells. An example of a Tomato^+^F4/80^+^*α*-SMA^+^ MMT cell (arrow) is shown in the lower panel. (**b**) Quantitative two-color flow cytometric analysis shows that more than 90% of F4/80+ cells express the Tomato reporter gene in both normal and UUO kidney. (**c**) Quantification data of flow cytometry shows that nearly 80% of *α*-SMA^+^ myofibroblasts co-express F4/80 in both day 3 and 7 UUO kidney, indicating that MMT cells (F4/80^+^*α*-SMA^+^ cells) account for the majority of the total *α*-SMA^+^ myofibroblast population. (**d**) Three-color flow cytometric analysis of cells isolated from the UUO kidney in which *α*-SMA^+^Tomato^+^ double-stained cells are then analyzed for expression of the F4/80 antigen. The majority of *α*-SMA^+^ myofibroblasts (65±3.9%) co-express both the Tomato transgene and the F4/80 antigen, indicating their origin in the macrophage lineage. Data represent results from eight animals. Scale bar, 20 *μ*M

We sought to establish the MMT process using lineage tracing. We used LysM-Cre/Rosa26-tdTomato mice in which myeloid cells and their progeny permanently express the Rosa26-Tomato (red fluorescence) reporter. Confocal microscopy and flow cytometry analysis revealed that almost all F4/80^+^ macrophages co-expressed Tomato in normal and sham-operated kidneys of LysM-Cre/Rosa26-tdTomato mice (Figures 3a and b). Similarly, Tomato expression in other organs (liver, heart, peritoneum) was also largely restricted to resident macrophages and was clearly separated from *α*-SMA staining of vascular cells (Supplementary Figure S1).

Examination of LysM-Cre/Rosa26-tdTomato mice in the UUO model by confocal microscopy showed co-expression of Tomato and F4/80 in most cells (Tomato^+^F4/80^+^) in the obstructed kidney (Figure 3a). In addition, many Tomato^+^F4/80^+^ cells co-expressed *α*-SMA (Figure 3a). This was also demonstrated by analysis with Z-stacked images of a Tomato+ *α*-SMA+ cell (Supplementary Figures S2 and S3). Flow cytometric analysis of the obstructed kidney showed that more than 90% of F4/80+ macrophages co-expressed Tomato (Figure 3b). In addition, approximately 22% of Tomato^+^ cells in the obstructed kidney expressed *α*-SMA and these Tomato^+^*α*-SMA^+^ cells accounted for approximately 75–80% of the *α*-SMA+ population (Figures 3c and d). Further analysis by three-color flow cytometry showed that Tomato^+^F4/80^+^*α*-SMA^+^ cells accounted for 65% of total *α*-SMA^+^ myofibroblasts (65±3.9%) within the fibrosing kidney (Figure 3d), indicating that the majority of Tomato^+^*α*-SMA^+^ cells retained F4/80 expression in day 7 UUO kidneys.

**Figure 4:**
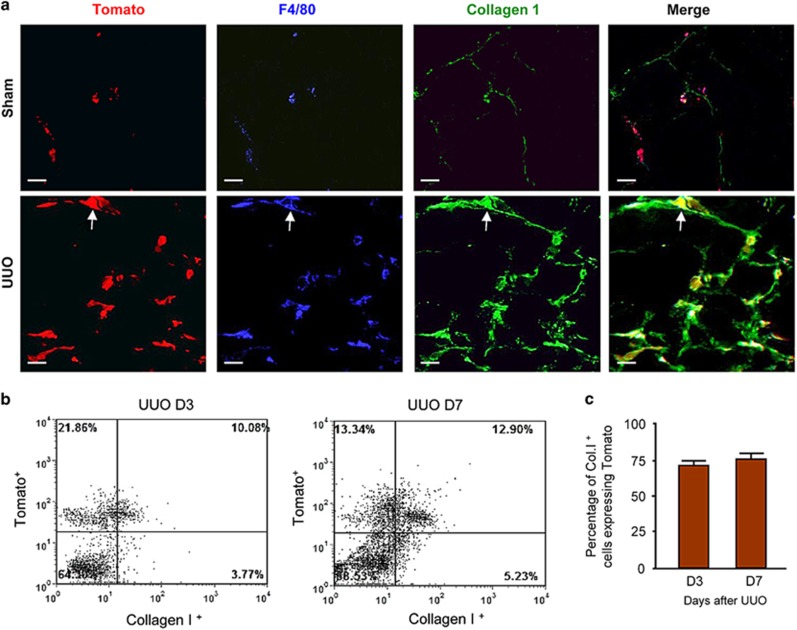
Fate mapping identifies collagen-producing MMT cells in the fibrotic kidney. Groups of eight LysM-Cre/Rosa26-tdTomato transgenic and littermate wild-type mice underwent unilateral ureteric obstruction (UUO) or sham surgery and were killed 7 days later. (**a**) Three-color confocal microscopy shows very occasional collagen I-producing Tomato^+^F4/80^+^ cells in sham-operated kidneys, whereas the majority of collagen I-producing cells are Tomato^+^F4/80^+^ macrophage lineage cells in the UUO kidney. Examples of collagen-producing Tomato^+^F4/80^+^ cells in the fibrosing kidney are indicated by arrows. (**b** and **c**). Three-color flow cytometric analysis and quantitative data demonstrate that the majority (70-80%) of col I^+^ cells in day 3 and 7 UUO kidneys co-express the Tomato reporter, demonstrating that they originate from the macrophage lineage. Data represent results from eight animals. Scale bar, 20 *μ*M

Production of collagen type I is a feature of active myofibroblasts in the fibrosing kidney. Confocal microscopy identified collagen I staining in the majority of Tomato^+^F4/80^+^ cells in the obstructed kidney (Figure 4a). Flow cytometric analysis confirmed this observation by detection of Tomato expression by 70% of collagen I^+^ cells on days 3 and 7 of the UUO model (Figures 4b and c).

**Figure 5:**
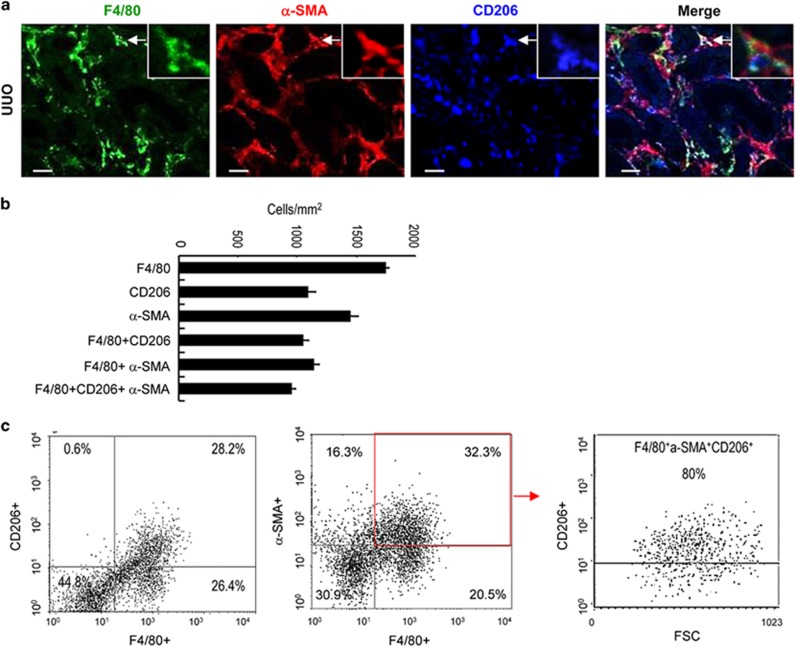
Macrophages undergoing transition have a predominant M2 phenotype in the UUO kidney. (**a**) Three-color confocal microscopy identifies *α*-SMA^+^F4/80^+^CD206^+^ triple-stained M2-type cells (arrows) in an area of interstitial fibrosis in day 7 UUO mouse kidney. (**b**) Quantitative analysis of single-, double- and triple-stained cell populations from the immunofluorescence staining for day 7 UUO mouse kidney (*n*=6). (**c**) Three-color flow cytometric analysis of cells isolated from day 7 UUO kidney. Approximately 50% of F4/80^+^ cells co-express CD206 in the UUO kidney. However, gating on *α*-SMA^+^F4/80^+^ double-stained cells identifies that approximately 80% of these cells express CD206, indicating enrichment of M2-type macrophages in this population. Scale bar, 20 *μ*M

The M2 marker, CD206, was examined in MMT cells in the UUO model. Triple staining of tissue sections identified that approximately 60% of F4/80^+^ macrophages expressed CD206 in this model, while 85% of F4/80^+^*α*-SMA^+^ MMT cells expressed CD206 (Figure 5). This was confirmed by flow cytometry, which showed that 80% of F4/80^+^*α*-SMA^+^ MMT cells expressed CD206 (Figure 5c).

### Myofibroblast accumulation and renal fibrosis is reduced by depletion of the myeloid lineage

**Figure 6:**
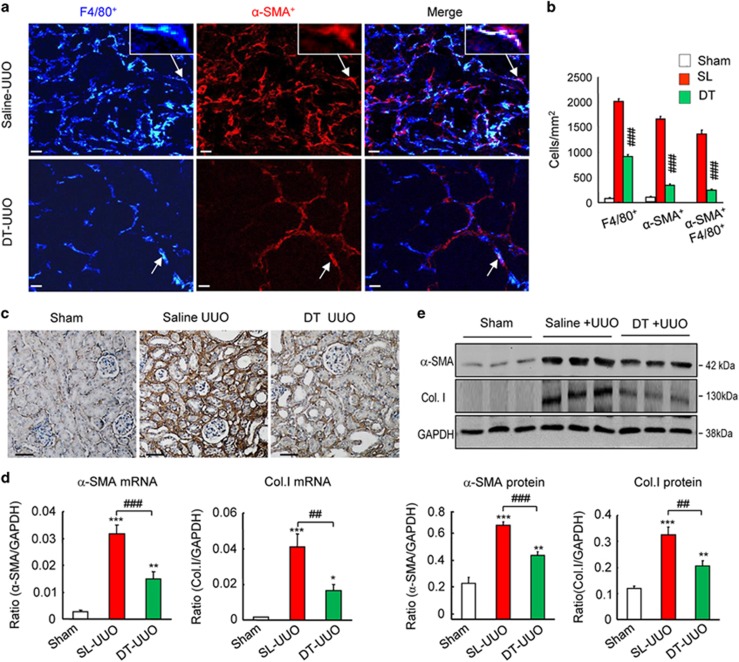
Conditional depletion of macrophages in LysM-Cre/DTR mice using diphtheria toxin (DT) attenuates the MMT response and renal fibrosis in obstructive nephropathy (UUO). (**a** and **b**) Results of three-color confocal immunofluorescence show that administration of DT reduces F4/80+ macrophages by 60% in the UUO kidney, leading to a dramatic reduction in the accumulation of F4/80^+^*α*-SMA^+^ cells and total *α*-SMA^+^ myofibroblasts. (**c**) Immunohistochemistry off collagen I staining. (**d**) Real-time PCR analysis of *α*-SMA and collagen I mRNA levels; (**e**) western blot analysis and quantification of *α*-SMA and collagen I protein levels. Each bar represents mean±S.E.M. for six mice. *P<0.05, **P<0.01, ***P<0.001 *versus* sham-control; ^##^*P*<0.01, ^###^*P*<0.001 saline-control or as indicated. Scale bar, 50 *μ*M

Lineage tracing based on the LysM-Cre promoter identified macrophages as a substantial source of myofibroblasts in the UUO model (Figures 3 and 4). One implication of this finding is that deletion of the LysM-Cre lineage would be predicted to prevent the infiltration of macrophages into the kidney and the subsequent transdifferentiation into MMT cells and therefore reduce renal fibrosis in the UUO model. To test this, we created LysM-Cre/iDTR mice, in which cells of the myeloid lineage express the human diphtheria toxin receptor. Administration of diphtheria toxin caused a 60% reduction in the accumulation of F4/80^+^ macrophages on day 7 UUO (Figures 6a and b). This macrophage depletion had a profound effect upon the accumulation of both total *α*-SMA+ myofibroblasts and F4/80^+^*α*-SMA^+^ MMT cells (Figures 6a and b). Immunohistochemistry, western blotting and PCR analysis demonstrated that macrophage depletion significantly reduced *α*-SMA and collagen I mRNA and protein levels in the obstructed kidney (Figures 6c–e).

## Discussion

Our recent study identified that bone marrow-derived macrophages can undergo myofibroblast transition *in vitro* and in experimental renal fibrosis via a process of MMT driven by the TGF-*β*/Smad3 signaling pathway.^17^ The current studies provide extensive new data to support this postulate based on: (i) identification of CD68^+^*α*-SMA^+^ myofibroblasts in areas of active fibrosis in patients with kidney disease; (ii) establishing that MMT cells have a predominant M2 phenotype in human kidney fibrosis; (iii) the use of myeloid lineage tracing to validate that macrophages can undergo conversion into a collagen I-producing myofibroblast in experimental renal fibrosis; and (iv) depletion of myeloid lineage cells prevented the appearance of MMT cells and substantially reduced myofibroblast accumulation and collagen deposition in experimental renal fibrosis.

Although myofibroblast differentiation and activation are recognized as central events in the pathogenesis of renal fibrosis, the origin of this myofibroblast population remains controversial.^7, 18^ A 1995 study identifying expression of fibroblast specific protein-1 (FSP-1) in tubular epithelial cells led to the concept of EMT as a source of myofibroblasts in renal fibrosis,^19^ although this hypothesis has been challenged in recent years due to findings from lineage tracing studies and a lack of persuasive clinical evidence.^5, 6, 7^ Therefore, we considered it is important to validate our previous study of the MMT process in renal fibrosis with both human data and lineage tracing data. The identification of significant numbers of CD68^+^*α*-SMA^+^ MMT cells in active fibrotic lesions in human disease, but their absence in acute inflammation and advanced sclerosis, provides strong support for the importance of MMT in the pathogenesis of renal fibrosis in human chronic kidney diseases and provides clinical relevance to the findings in the animal studies. The detection of MMT cells in rapidly progressive forms of glomerulonephritis is not surprising given the prominent macrophage infiltrates seen in these conditions, but MMT cells detected in patients with IgA nephropathy reveal MMT as a common process during the development of progressive renal fibrosis. Thus, MMT may be an important mechanism in the progression from acute macrophage-rich inflammation towards active fibrosis. The low percentage of α-SMA^+^ myofibroblasts co-expressing macrophage markers in advanced sclerotic lesions presumably reflects a completion of this transition process.

The lineage tracing studies using the well-characterized LysM-Cre promoter to drive Tomato expression in myeloid cells provided a robust demonstration of the MMT process using both confocal microscopy and flow cytometry in experimental renal fibrosis. These findings extend our previous studies using *ex vivo* dye labeling of isolated macrophage populations and GFP+ bone marrow chimeric mice.^17^ In addition, while the approach of flow cytometry to measure collagen I production by fibroblasts has been questioned recently,^20^ the demonstration that Tomato^+^ cells produced collagen I in the UUO model is consistent with our previous study in which CD11b^+^ macrophages infiltrating into the obstructed kidney markedly up-regulate collagen I and III mRNA levels. It is noteworthy that Tomato^+^ F4/80^+^ *α*-SMA^+^ MMT cells account for more than 60% of the total number of *α*-SMA^+^ cells, demonstrating the potential importance of macrophage-derived myofibroblasts.

In addition to using lineage tracing to identify a macrophage origin for a substantial proportion of collagen-producing myofibroblasts in renal fibrosis, we also performed the reciprocal experiment in which the functional requirement of myeloid-derived myofibroblasts in the development of renal fibrosis was demonstrated by depleting cells of this lineage in LysM-Cre/DTR mice. Although a reduction in myofibroblast accumulation and collagen deposition has been described previously with different macrophage depletion strategies in the UUO model,^21, 22^ the current studies provide the critical missing link by establishing that macrophages exert a pro-fibrotic effect by directly transforming into myofibroblasts via the MMT process.

The presence of MMT may reflect disease activity and fibrotic progression in human kidney disease and it may also explain the notion that the density and heterogeneity of the macrophage infiltrate determine the fate of renal inflammation and fibrosis.^23, 24^ It is of note that infiltration of M2 macrophages positively correlates with the progression of renal fibrosis in human kidney diseases.^25, 26^ In this setting, the present study identified that the large majority of MMT cells in human kidney fibrosis express the M2 marker, CD206. This confirms the relevance of CD206 expression by MMT cells in the UUO model, confirms our previous studies indicating that M2 macrophages had a greater capacity to undergo MMT compared with M1 macrophages^17^ and gives support to other studies demonstrating that bone marrow-derived M2 macrophages are highly proliferative and pro-fibrotic.^27, 28^ Based upon the present findings and our recently published work, we propose a new paradigm in renal fibrosis in which infiltrating bone marrow-derived blood monocytes can differentiate into M1 pro-inflammatory macrophages and cause injury to the kidney. Consequently, a repair process driven by TGF-*β*1 induces a switch of M1 macrophages towards an M2 pro-fibrotic phenotype.^29, 30, 31, 32, 33^ A key component in this repair process is TGF-β1/Smad3-driven MMT,^17, 34, 35^ which induces the transition of M2 macrophages into collagen-producing *α*-SMA^+^ myofibroblasts which contribute to the excessive deposition of extracellular matrix occurring during renal fibrosis.

The results of this study are consistent with recent experiments identifying a significant contribution of bone marrow-derived fibroblasts in mouse models of renal fibrosis.^36^ Indeed, it may be the case that these bone marrow-derived fibroblasts are the same population identified in our lineage tracing studies; however, this remains to be established. In addition, recent studies have shown a role for adiponectin/AMPK and JAK3/STAT6 signaling pathways in the development of experimental renal fibrosis and in collagen production by cultured mouse monocytes,^37, 38^ providing new potential therapeutic targets in addition to TGF-*β*/Smad3 signaling.

In conclusion, we have shown that macrophages can transdifferentiate into collagen-producing *α*-SMA^+^ myofibroblasts during renal fibrosis in human and experimental kidney disease. Targeting the MMT process may represent a novel therapeutic approach for the treatment of fibrotic renal diseases.

## Materials and Methods

### Patient samples

Human biopsy tissue samples were collected from the Department of Anatomical and Cellular Pathology, Chinese University of Hong Kong. Biopsies were from patients with minimal change disease (*n*=15), IgA nephropathy (*n*=21) and rapidly progressive glomerulonephritis (*n*=22). The uninvolved tissue from nephrectomy specimens performed for renal tumors was used as normal controls (5). MMT cells were detected by two-color confocal microscopy with monoclonal antibodies to macrophage (CD68) and myofibroblast (*α*-SMA) markers. All renal biopsies were examined for the presence of three types of pathological lesions: (a) early or acute inflammation featuring mild histological damage with abundant CD68^+^ macrophages but few *α*-SMA^+^ myofibroblasts; (b) active tissue fibrosis where an approximately equal number of CD68^+^ macrophages and *α*-SMA^+^ cells were detected; and (c) advanced sclerosis with severe tissue damage featuring numerous *α*-SMA^+^ cells and few CD68^+^ macrophages. The numbers of CD68^+^ cells, *α*-SMA^+^ cells and CD68^+^*α*-SMA^+^ cells were scored in these lesions for each renal biopsy tissue. All experiments were approved by Joint Chinese University of Hong Kong-New Territories Eater Cluster Clinical Research Ethics Committee.

### Animals and animal models

LysM-Cre mice (stock number 004781) and Cre-dependent tdTomato reporter transgene mice (stock number 007914, referred to as Rosa26-tdTomato mice), both on the C57/Bl6 background, were purchased from the Jackson Laboratory (Bar Harbor, ME, USA). The mouse strains were intercrossed to generate LysM-Cre/Rosa26-tdTomato mice. Groups of eight LysM-Cre/Rosa26-tdTomato mice and their littermate control mice underwent UUO or sham surgery and were killed 7 days later as previously described.^39^

In the macrophage depletion study, macrophages were conditionally deleted from LysM-Cre/DTR mice by intraperitoneal injection of diphtheria toxin as previously described.^40^ UUO nephropathy was performed by ligation of the left ureter and mice were killed 7 days later. Groups of 6–8 mice of both genders, aged 8–10 weeks were studied. The experimental protocols were approved by the Institutional Animal Experimentation Ethics Committee of The Chinese University of Hong Kong.

### Immunofluorescence and confocal image analysis

Immunofluorescence and confocal microscopy were performed on frozen sections. Macrophages were labeled with rat anti-mouse F4/80 (Serotec Ltd, Oxford, UK) or FITC-conjugated mouse anti-human CD68 (DAKO, Carpinteria, CA, USA), while myofibroblasts were detected with the Cy3-labeled mouse anti-mouse/human *α*-SMA (Sigma, St. Louis, MO, USA). Other antibodies used include goat anti-collagen I (Southern Tech, Birmingham, AL, USA), FITC-labeled rat anti-mouse CD206 (Serotec Ltd), Cy5-labeled goat anti-rabbit antibody (Invitrogen, Carlsbad, CA, USA) and Alexa555-labeled donkey anti-goat antibody (Invitrogen). Positive signals were examined using a fluorescent microscope (Axioplan2 imaging; Carl Zeiss, Oberkoche, Germany) or a confocal microscope (LSM 510 META, Carl Zeiss) and single-, double- or triple-positive cells were counted in 10 high-power fields ( × 40) per section and expressed as cells per square millimeter.

### Flow cytometric analysis

Single cells were isolated from both normal and fibrotic kidneys using enzyme-digestion and analyzed by flow cytometry as previously described.^17^ After permeabilization with Fixation/Permeabilization buffer kit (eBioscience, San Diego, CA, USA), cells were incubated with FITC-conjugated rat anti-mouse F4/80 (eBioscience) followed by Cy5-conjugated goat anti-rat IgG (Millpore), PE-conjugated anti-*α*-SMA (R&D, Minneapolis, MN, USA), FITC-labeled rat anti-mouse CD206 (Serotec). Collagen I was stained with rabbit anti-mouse collagen I (Millpore) followed by the Cy5-labeled goat anti-rabbit IgG (Invitrogen). Cells stained with isotype-matched irrelevant control antibodies and unstained cells were used as negative controls. Cells were detected by a FACS Calibur flow cytometer (BD Biosciences, San Jose, CA, USA) and analyzed by Cellquest software (BD Biosciences).

### Immunohistochemistry

Immunostaining was performed on 3-*μ*m paraffin sections using a microwave-based antigen retrieval technique.^41^ The antibodies utilized in the present study included collagen I (Southern Biotech), *α*-SMA (Sigma) and F4/80 (Serotec).

### Real-time PCR and western blot analysis

Total RNA was isolated from kidney tissue by using the RNeasy Isolation Kit (Qiagen, Inc., Valencia, CA, USA) according to the manufacturer's instructions. Collagen I and *α*-SMA were quantitatively analyzed at the mRNA levels by real-time PCR and at the protein levels by western blot analysis as previously described.^41, 42, 43^

### Statistical analysis

Data are expressed as the mean±S.E.M. and analyzed using one-way analysis of variance (ANOVA), followed by Tukey's *post hoc* tests using GraphPad Prism 5. Numbers of CD68^+^*α*-SMA^+^ MMT cells from acute and active lesions (*n*=32) of renal biopsy samples were correlated with *α*-SMA^+^ cells using the Spearman correlation analysis.
