# Supplementary figures and images for: Inflammatory macrophages can transdifferentiate into myofibroblasts during renal fibrosis
Source: Cell Death Dis. 2016 Dec 1;7(12):e2495–. doi: 10.1038/cddis.2016.402 (PMC5261004; doi:10.1038/cddis.2016.402)

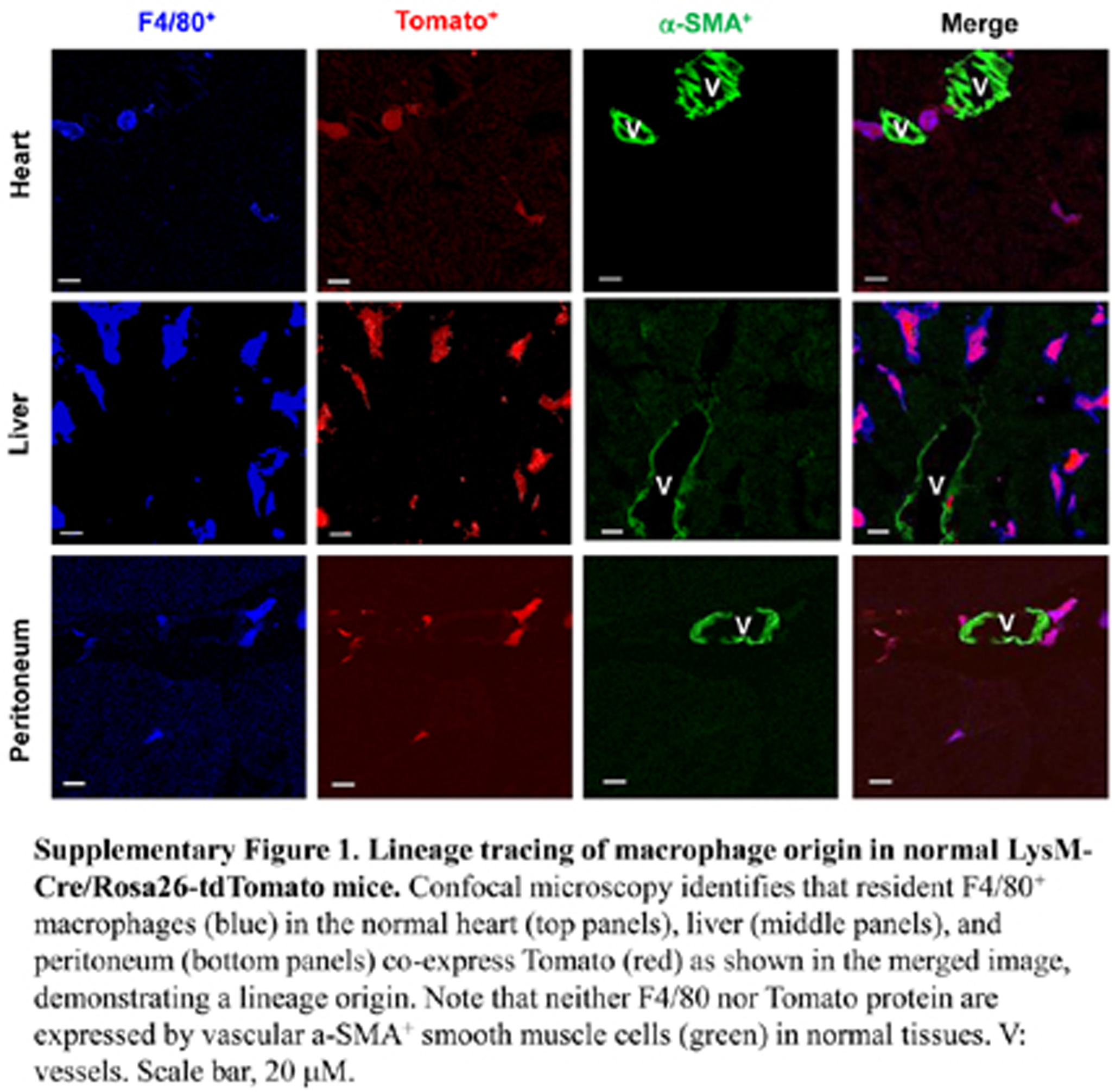

Supplement: Supplementary Figure 1 [file cddis2016402x1.tif]

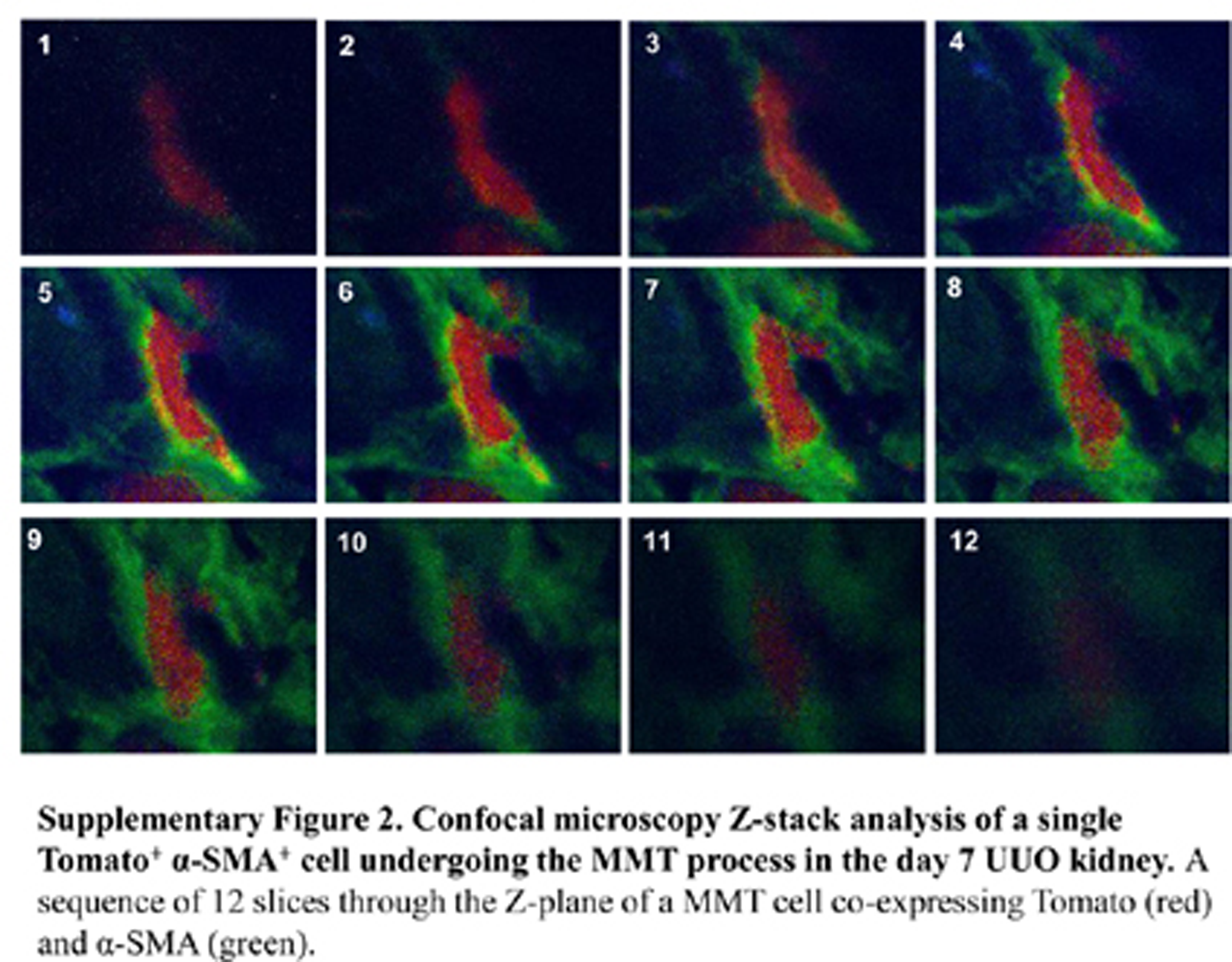

Supplement: Supplementary Figure 2 [file cddis2016402x2.tif]
